# Chest resurfacing with a reverse abdominoplasty flap for invasive breast cancer recurrence

**DOI:** 10.1080/23320885.2019.1605293

**Published:** 2019-04-23

**Authors:** Pietro G. di Summa, Clara Schaffer, Sherilyn K. Tay, Eva Weiler-Mithoff

**Affiliations:** a Department of Plastic, Reconstructive and Hand Surgery, University Hospital of Lausanne (CHUV), Lausanne, Switzerland;; b Canniesburn Plastic Surgery Unit, Glasgow Royal Infirmary, Glasgow, United Kingdom

**Keywords:** Breast cancer recurrence, chest resurfacing, radiotherapy, Reverse Abdominoplasty flap

## Abstract

We present a patient with recurrent breast cancer requiring massive resection of the upper chest. Management included a reverse abdominoplasty flap to resurface the anterior chest wall, with acceptable aesthetic outcomes, and adjuvant chemotherapy. RA is a simple and versatile coverage option in patients with high risk of disease recurrence.

## Introduction

Breast cancer is one of the most frequently encountered indication for chest wall reconstruction with a frequency from 20 to 40% depending on series [1,2]. Whereas the overall 10-year survival rate of patients with primary breast cancer without metastasis at diagnosis is 86% [3], the reported mortality rate after local recurrence is 63.7% after 10 years, and death occurs on average 2.6 years after the recurrence [4]. Indeed, treatment of a recurrence must be aggressive to prevent further relapse and death.

The reverse abdominoplasty (RA) technique has been typically described for reshaping the upper abdominal contour when there is predominantly upper abdominal laxity [5,6], but may serve as a reconstructive procedure to cover soft tissue defects of the trunk [7] after tumour resection (2).

Considering the extensive use of latissimus dorsi pedicled flaps and free flaps for breast reconstruction or chest resurfacing, the literature is very scarce regarding the use of the reverse abdominoplasty for chest resurfacing after oncologic procedures [8–10]. In fact, RA is rarely adopted but may be a simple and effective way to resurface chest defects, especially when more complex solutions have been already used or may not be tolerated by patients that are systemically unstable. Indeed, it could be also used as effective temporary solution for coverage after local disease control. Previous reports showed good aesthetic and functional results while guaranteeing few complications when compared to more complex procedures such as pedicled flaps or free tissue transfer [5].

Little is known about the reliability to perform RA when the chest wall has been previously irradiated [11], especially in presence of relevant defects.

We present a case of a patient who required bilateral mastectomies and reconstruction with latissimus dorsi flap and adjuvant radiotherapy, who subsequently presented with extensive recurrence of the inflammatory breast cancer requiring extensive resection of both the reconstructions and native skin within the previous radiotherapy field. A reverse abdominoplasty flap covered the resultant thoracic soft tissue defect and further adjuvant chemotherapy completed the oncologic management.

## Case report

A 49 year-old BRCA-1 positive underwent neoadjuvant chemo and radiotherapy followed by bilateral mastectomies and latissimus dorsi reconstructions. Of note, in the previous 15 years, she had already presented three triple-negative breast cancers in both breasts, treated by multiple wide local excisions and radiotherapy. Unfortunately, 3 months after the bilateral mastectomies, she developed a local recurrence at the level of the right reconstructed breast, extending towards the midline, without evidence of distant metastasis. Multiple wide local excisions of the clinically involved mastectomy flaps were performed but did not achieve clear margins. Excision of the breast with recurrence was therefore planned with reconstruction using a bipedicled DIEP to allow replacement of the volume and shape.

However, multiple mapping biopsies were performed to determine the extent of disease and the disease was found to be extend on to, but not entirely, the contralateral non-irradiated breast. The entire radiotherapy field was therefore thought to have obliterated normal anatomic barriers to tumour spread and would therefore require complete excision of both irradiated skin and reconstructions. Given the likely resulting defect based on the biopsies, the bipedicled DIEP was therefore insufficient to reconstruct the defect and allow primary donor defect closure and the decision was made to reconstruct with a reversed abdominal flap.

**Figure 1.**
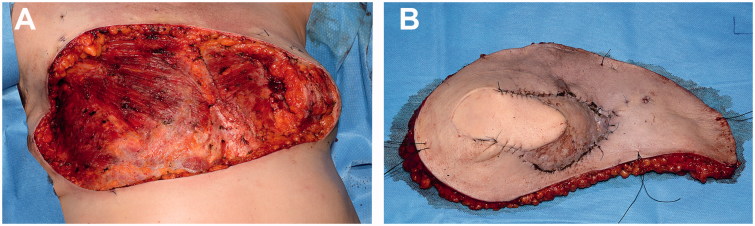
Intraoperative images. (A) Extensive thoracic defect after complete surgical excision of breast cancer recurrence (lateral view). (B) Resected tumour.

**Figure 2.**
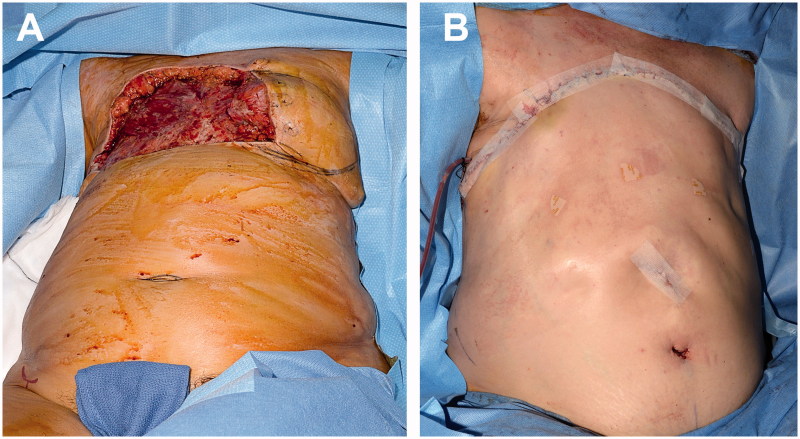
Intraoperative images. (A) Thoracic defect before coverage (anterior view). (B) Immediate result after defect coverage with a reverse abdominoplasty flap.

After a radical R0 resection (Figure 1), we performed a reconstruction with a reverse abdominoplasty flap to reconstruct the remaining 30 × 18 cm soft tissue defect (Figure 2).

Abdominal flap was raised from cranial to caudal, over the anterior rectus sheath. Initial central undermining was followed by progressively lateral undermining, sparing main perforators. Given the extent that the abdominal flap had to be mobilised, the umbilicus required resitting and therefore a second caudal incision at the level of umbilicus allowed better infraumbilical flap mobilisation was made and dissection continued using a lit retractor through this incision. Multiple quilting sutures (Vycril 2.0, Ethicon) were used to allow tension free closure of the defect.

**Figure 3.**
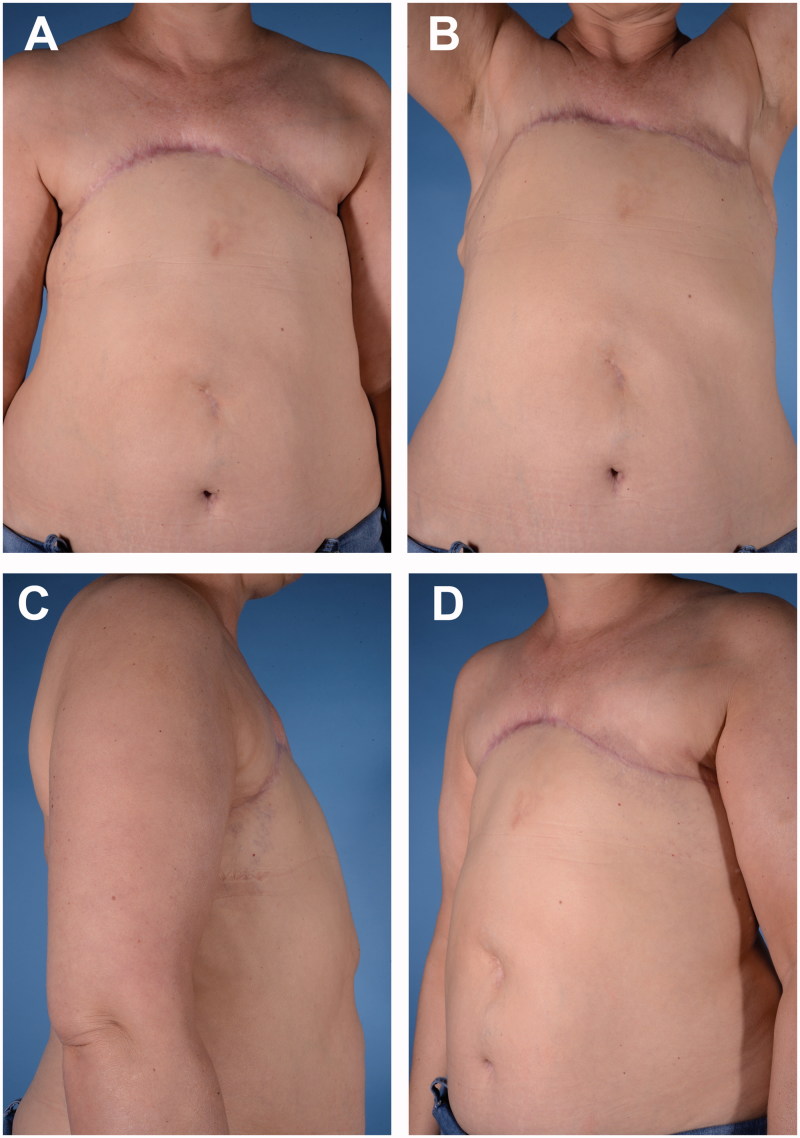
Postoperative aesthetic outcome at 11 months follow-up. (A) Anterior view. (B) Anterior view with arms lifted up. (C) Right lateral view. (D) Left oblique view.

The abdominal flap was monitored intraoperatively for bleeding form the edges; the lumbar segmental arteries network provided sufficient vascular supply, together with the spared perforators. The abdominal flap healed uneventfully allowing delivery of adjuvant chemotherapy 2 months after chest resurfacing was performed. No flap-related nor donor site complications were witnessed. Patient showed effective chest resurfacing and satisfactory functional results at 11 months follow-up, without evidence of further local or distant recurrence and fair aesthetic result (Figure 3). Patient produced informed consent, including permission for photographic and video documentation.

## Discussion

After conventional mastectomy, autologous flap transfer allows reconstruction of both breast volume and shape. When dealing with a poorly controlled tumour, most commonly inflammatory breast cancer and sarcoma, the concept of breast reconstruction shifts to chest resurfacing. Priority is to perform complete oncological resection to prevent recurrence and increase survival of the patient, and to achieve coverage of the defect with a simple and effective reconstructive procedure. There are many options, depending on the nature, size of the defect, location, prognosis of the tumour and availability of recipient vessels in case of free flap reconstruction [12]. In the majority of patients, regional myocutaneous flaps, most commonly the latissimus dorsi, pectoralis major and transverse rectus abdominis pedicled flaps, provide sufficient soft tissue coverage. Free flaps should be also considered if recipient vessels are available and if patients are fit for a longer and more challenging procedure. However, prior radiotherapy and local invasion could jeopardise vascularisation of both local flaps and potential receiving vessels for microvascular anastomoses, significantly challenging the reconstruction with longer operative times and higher failure risks [1]. In such cases the reverse abdominoplasty can provide effective soft tissue without the need for free tissue transfer [8].

Anatomically, the vascular supply of the abdominal wall is divided into three areas. The central abdomen is supplied by the superior epigastric arteries, the inferior abdomen by the deep and superficial inferior epigastric arteries, deep and superficial circumflex iliac arteries and the superficial external pudendal artery. Finally, the intercostal, subcostal and lumbar segmental arteries supply the peripheral abdomen [13]. In the reverse abdominoplasty technique, the peripheral vascularisation provides a reliable blood supply to the flap despite wide undermining. Quilting sutures are used to reduce tension on skin edges and minimise dead space. Here we show that the reverse abdominoplasty is a versatile, simple and reliable solution for resurfacing the anterior chest wall, with acceptable aesthetic outcome [7,8,11].

Ideal indications are represented by patients with a high chance of disease recurrence (e.g. recurrent breast cancer), in whom you may want to preserve salvage options or in whom other options have already been utilised such as in this case and in patients in poor general condition, requiring a shorter surgical procedure and who would not be candidates for more complex or microsurgical reconstructions. In our case, the patient benefited from a less invasive and quickly healing procedure, (instead of free abdominal tissue transfer after which we would have had difficulty closing the donor site) which allowed her to receive adjuvant chemotherapy shortly after reconstruction. The abdominal flap was raised, as described, uneventfully, and showed efficient vascularity, despite previous irradiation of upper chest, covering the extensive defect. This minimised reconstruction related-risks of delaying adjuvant chemotherapy. As a drawback, this technique requires sufficient abdominal laxity and is not suitable for combined soft tissue and skeletal defects, since reconstruction should preserve stability and function of the chest wall [1]. In case of further recurrence or distal flap necrosis, flap could be re-advanced, as showed in previous reports [8].

The reverse abdominoplasty needs to be remembered as simple and effective technique to provide fast coverage of extensive soft tissue defects of the chest wall, despite previous radiotherapy of the chest and when dealing with aggressive tumour or recurrences requiring extensive resection and early coverage, in patients who may require adjuvant treatment.
